# The validity of test-negative design for assessment of typhoid conjugate vaccine protection: comparison of estimates by different study designs using data from a cluster-randomised controlled trial

**DOI:** 10.1016/S2214-109X(25)00056-7

**Published:** 2025-04-16

**Authors:** Shuo Feng, Yiyuan Zhang, Farhana Khanam, Merryn Voysey, Virginia E Pitzer, Firdausi Qadri, John D Clemens, Andrew J Pollard, Xinxue Liu

**Affiliations:** a Oxford Vaccine Group, Department of Paediatrics, University of Oxford, Oxford, UK; b National Institute for Health and Care Research Oxford Biomedical Research Centre and Oxford University Hospitals NHS Foundation Trust, Oxford, UK; c School of Public Health, Tongji Medical College, Huazhong University of Science and Technology, Wuhan, China; d International Centre for Diarrheal Disease Research, Bangladesh, Dhaka, Bangladesh; e Department of Epidemiology of Microbial Diseases and Public Health Modelling Unit, Yale School of Public Health, New Haven, CT, USA; f International Vaccine Institute, Seoul, South Korea; g UCLA Fielding School of Public Health, Los Angeles, CA, USA; h Vaccine Innovation Centre, Korea University School of Medicine, Seoul, South Korea

## Abstract

**Background:**

Typhoid fever remains a substantial public health challenge in low-income and middle-income countries. By 2023, typhoid conjugate vaccines (TCVs) had been introduced in six countries globally, with more than 50 million doses distributed. Now that TCVs are being deployed, there is a need for observational studies to assess vaccine effectiveness in the field. We aimed to evaluate the validity of different observational study designs in estimating vaccine protection.

**Methods:**

We compared different observational and experimental study designs for assessing vaccine effectiveness by re-analysing data from the TyVAC Bangladesh trial, a participant-blinded and observer-blinded cluster-randomised controlled trial done in Mirpur, Dhaka, Bangladesh. 150 geographical clusters were randomly assigned (1:1) to receive either TCV or Japanese encephalitis vaccine. Eligible children aged 9 months to 15 years were offered a single dose of the vaccine randomly assigned to their cluster of residence, and baseline vaccination was done between April 15 and May 15, 2018. We compared estimates of vaccine effectiveness from the cluster-randomised controlled trial analysis—which assessed the risk of blood-culture-confirmed typhoid fever among recipients of TCV versus recipients of Japanese encephalitis vaccine—with estimates from cohort study and test-negative case–control study design (TND) analyses, which compared recipients of TCV with non-vaccinees in the 75 geographical clusters where TCV was administered. We further conducted negative-control exposure (NCE) and negative-control outcome (NCO) analyses as bias indicators.

**Findings:**

41 344 (67%) of 62 025 age-eligible children in the study area received the TCV or Japanese encephalitis vaccine during the baseline vaccination campaign. Among the 62 025 age-eligible children, 5582 blood-culture specimens were collected by passive surveillance, including 2546 (46%) specimens from the 75 TCV clusters. The estimated vaccine efficacy was 89% (95% CI 81–93) in the cluster-randomised controlled trial analysis, 79% (70–86) by the cohort design, 88% (79–93) by the TND when pan-negatives were used as test-negative controls, and 90% (75–96) by the TND when specimens positive for pathogens other than *Salmonella enterica serotype* Typhi were used as test-negative controls. Using NCE analysis, Japanese encephalitis vaccination was associated with an increased risk of typhoid fever compared with non-vaccinees in the 75 Japanese encephalitis clusters in the cohort design (incidence rate ratio 1·98 [95% CI 1·56–2·52]), but no significant association between Japanese encephalitis vaccination and typhoid fever was found with the TND. Similarly, an increased risk of non-typhoid infections was observed in the cohort NCO analyses when comparing vaccinees with non-vaccinees in both Japanese encephalitis vaccine clusters and TCV clusters, but not in the TND NCO analyses.

**Interpretation:**

Our findings suggests that the TND provides reliable estimates of TCV effectiveness, whereas the cohort design can bias vaccine effectiveness estimates, possibly due to unmeasured confounding effects, such as health-care-seeking behaviours. NCE and NCO approaches are useful tools for identifying such biases.

**Funding:**

The Bill & Melinda Gates Foundation

## Introduction

The disease burden due to typhoid fever, caused by *Salmonella enterica* serotype Typhi (*S* Typhi), remains high in low-income and middle-income countries with poor sanitation and limited access to clean water.^1^, ^2^, ^3^ The development and introduction of safe and effective typhoid vaccines has become a worldwide priority in controlling typhoid.^4^ The first typhoid conjugate vaccine (TCV) was prequalified by WHO in 2017. In 2019, Pakistan became the first country to introduce a TCV into its national immunisation programme with support from Gavi, the Vaccine Alliance. By the end of 2024, three TCVs were prequalified by WHO, and six countries had introduced a TCV, with more TCV candidates in the pipeline for prequalification.^5^

**Research in context**

**Evidence before this study**

We searched PubMed with no language restrictions from database inception to May 24, 2024, using the terms “typhoid conjugate vaccine” AND (“test-negative design” OR “case control” OR “cluster randomised controlled trial”), and found 39 publications. Although a methodology study using individually randomised controlled trial data showed good consistency in vaccine protection estimated by the test-negative case–control study design (TND) compared with results from the original trial, none of the four identified cluster-randomised studies on typhoid conjugate vaccine (TCV) extended the analysis to validate the TND or cohort study designs.

**Added value of this study**

This study showed a high degree of concordance between vaccine protection estimates from the TND and the cluster-randomised controlled trial analyses. By contrast, the cohort study design underestimated vaccine protection compared with the cluster-randomised controlled trial. Bias indicator analyses using a negative control exposure (NCE) and a negative control outcome (NCO) showed distorted associations in the cohort design, whereas there was no evidence of bias in the TND analyses.

**Implications of all the available evidence**

The results of our study strongly support the use of the TND for monitoring TCV effectiveness after introduction. Accurate vaccine efficacy estimates are crucial for the strategic planning of TCV programmes. Both TND and cohort studies should consider NCE or NCO as bias indicators when possible. Results from cohort designs should be interpreted cautiously when unmeasured confounders exist.

Similar vaccine efficacy of a TCV against blood-culture-confirmed typhoid fever was reported from randomised controlled trials in Nepal, Malawi, and Bangladesh, ranging from 79% to 85% in children aged from 9 months up to 16 years.^6^, ^7^, ^8^, ^9^ However, the effectiveness of TCVs estimated by observational studies after vaccine introduction varies between settings and study designs.^10^, ^11^, ^12^, ^13^ Although vaccine effectiveness might genuinely differ between study populations, the primary driver of disparities in reported efficacy across studies is likely to be the variation in study designs.

Monitoring vaccine effectiveness after vaccine introduction is crucial to evaluate vaccination programmes in the long term. Observational study designs play a key role in providing evidence of vaccine performance after implementation. The test-negative case–control study design (TND) is a popular approach to monitoring vaccine effectiveness and has been widely implemented to assess vaccine programmes.^14^ In a TND study, participants are enrolled among people presenting with similar symptoms, and cases and controls are defined by the results of diagnostic testing for the pathogen of interest (ie, cases test positive for the pathogen of interest, and controls test negative). Compared with the traditional case–control study (in which controls are not restricted to people seeking health care and had diagnostic testing), TND studies can theoretically reduce confounding caused by possible differences in health-care-seeking behaviours between cases and controls.^15^

Methodological research has shown promising results when comparing vaccine effectiveness estimated by the TND with efficacy estimated by randomised controlled trial as the gold standard.^12^, ^16^ Notably, these studies were conducted using datasets from individually randomised controlled trials and included participants who received a control vaccine or placebo as the substitute for the unvaccinated population in a real-world TND study. As a result, the theoretical advantage of the TND in reducing confounding due to differences in health-care-seeking behaviours between unvaccinated and vaccinated populations cannot be evaluated. Unlike individually randomised controlled trials, cluster-randomised controlled trials randomly assign clusters of participants, offering the randomised intervention to all eligible individuals within each cluster. Similar to real-world vaccine campaigns, participants can choose whether to be vaccinated or not, resulting in two groups of people with potentially different health-care-seeking behaviours in each cluster. Further research using simulated cluster-randomised controlled trials aimed to fill the knowledge gap and showed consistent estimates of vaccine effectiveness in the TND studies and the cluster-randomised controlled trials.^17^, ^18^ To our knowledge, there is no study using real data from cluster-randomised controlled trials to confirm these findings.

We aimed to evaluate the validity of the TND in estimating the effectiveness of a TCV using data from the TyVAC Bangladesh cluster-randomised controlled trial.^6^ The odds of vaccination were compared between those testing positive for *S* Typhi (cases) and those testing negative for *S* Typhi (controls) in the TCV clusters, which consisted of TCV vaccinees and non-vaccinees (ie, all those in the 75 geographical cluster where TCV was administered, including children who did and those who did not receive the vaccine), to create a situation that closely mirrors real-world settings (in which eligible people can decide to take a vaccine or not once the vaccine is available). In addition, vaccine effectiveness estimated by the cohort design in the TCV clusters was also assessed and compared with the TND and cluster-randomised controlled trial.

## Methods

### Study design and participants

TyVAC Bangladesh is a participant-blinded and observer-blinded cluster-randomised controlled trial^6^ conducted in Mirpur, Dhaka, Bangladesh, an urban slum with an approximate population of 200 000 people. The study area was divided into 150 geographical clusters, which were then randomly assigned (1:1) to receive TCV (Typbar TCV, Bharat Biotech International, Hyderabad, India), or SA-14-14-2 Japanese encephalitis vaccine (Chengdu Institute of Biological Products, Chengdu, China) as the control. Eligible children aged 9 months to 15 years were offered a single dose of the vaccine randomly assigned to their cluster of residence, and baseline vaccinations were administered between April 15 and May 15, 2018. Considering the high migration within the study area, three catch-up vaccination campaigns were conducted at 6-month intervals. The vaccine coverage rate was similar between the TCV and Japanese encephalitis vaccine groups (both 65%).^6^ Vaccination information for each individual was recorded in an electronic data capture system during vaccination campaigns. Passive surveillance for enteric fever started in February, 2018, before the baseline vaccination campaign. Vaccination information captured during campaigns was linked with passive surveillance data via study IDs. Participants in the study area who presented at any of the eight study clinical facilities with at least 2 days of fever or with an axillary temperature of at least 38°C were recruited into the trial after providing informed consent, regardless of their vaccination status. A blood specimen was collected from each resident presenting with fever, and blood culture results were reported as negative or positive for *S* Typhi, *Salmonella enterica s*erotype Paratyphi (*S* Paratyphi), non-typhoidal *Salmonella*, and other pathogens. The surveillance system was suspended on March 15, 2020, owing to the COVID-19 pandemic. The data collected between baseline vaccination and March 15, 2020, were used in this study (study timeline is shown in appendix 1 p 8). Febrile visits occurring within a 14-day window after discharge from a previous febrile episode were considered one febrile event. Full study design details for the TyVAC Bangladesh study, including sample size calculation and detailed methodology for blood specimen culture, have been described previously.^6^ The TyVAC Bangladesh trial received ethical approval from the research and ethical review committee of the International Centre for Diarrhoeal Disease Research, Bangladesh, as well as the institutional review board of Oxford University, Oxford, UK. Written informed consent was obtained from all participants, including parent or guardian consent for all those younger than 16 years and participant assent for those aged 11–16 years, in the original trial. Sex data were reported by the parent or guardian on behalf of the participants. TyVAC Bangladesh is registered with ISRCTN, ISRCTN11643110.

### Procedures

**Figure 1:**
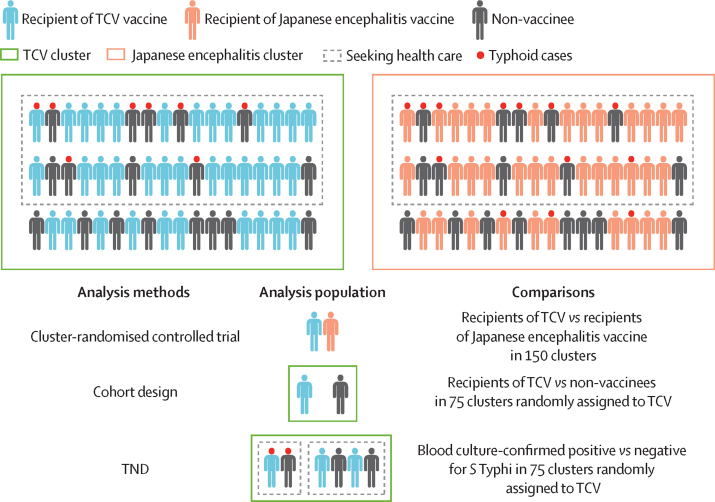
Study designs *S* Typhi=*Salmonella enterica* serotype Typhi. TCV=typhoid conjugate vaccine. TND=test-negative case–control study design.

In this study, we compared vaccine protection estimated by three analysis methods: cluster-randomised control trial, cohort design, and TND (figure 1). The primary analysis focused on a closed cohort of participants who were aged 9 months to 15 years at the baseline vaccination campaign to simulate a situation in which there was only one vaccination campaign, for all analysis methods. We also conducted a sensitivity analysis by including all the children who were age-eligible at any of the four vaccination campaigns, covering both children living in the study area at baseline and those who aged or migrated into the study area between April 15, 2018, and March 15, 2020.

### Statistical analysis

Detailed information on the statistical analysis is in appendix 1 (p 3). In brief, in the cluster-randomised controlled trial analysis of all 150 clusters, we included all the children who received one dose of TCV or Japanese encephalitis vaccine, to estimate vaccine effectiveness, as the gold standard. For the primary cohort analysis, we emulated a hypothetical target trial among residents in the 75 geographical clusters in which TCV was administered, including all children who met the eligibility criteria at the baseline vaccination campaign, regardless of their vaccination status.^19^, ^20^, ^21^ These children were grouped into vaccinees and non-vaccinees based on their vaccination status at the baseline campaign. The inverse probability of censoring weighting was used to control the potential effect of censoring in the cluster-randomised controlled trial and cohort analyses.

In the TND primary analysis, all the blood specimens from age-eligible children at the baseline vaccination campaign in the 75 TCV clusters were included. Test-positive specimens were defined as those that were positive for *S* Typhi, and two definitions were applied for test-negative specimens: positive for pathogens other than *S* Typhi (including positive for *S* Paratyphi, non-typhoidal *Salmonella,* and other pathogens) and no growth (pan-negative), to evaluate the effect of blood culture sensitivity on the TND results. 127 specimens with cultures that were positive for other pathogens were identified as contaminants, and we conducted two analyses by including and excluding them as test-negative controls.

To validate the theoretical advantage of the TND over a traditional matched case–control design in reducing biases, 1000 matched case–control studies were simulated for comparison. We further used a negative control exposure (NCE) and a negative control outcome (NCO) approach to assess potential biases.^22^, ^23^, ^24^ NCE approaches have been increasingly used in epidemiology studies, including those assessing vaccine effectiveness, to identify confounding, selection, and measurement biases.^25^ An NCE method tests an exposure that is susceptible to the same sources of bias as the primary exposure but does not causally affect the outcome. Similarly, an NCO method uses an outcome that is not plausibly affected by the exposure of interest. In our NCE analysis, the cohort and TND analyses in TCV clusters were repeated in the 75 geographical clusters in which Japanese encephalitis vaccines were administered (including all children who received and did not receive this vaccine), taking Japanese encephalitis vaccination as the negative exposure. For the NCO analysis, we replaced the outcome of typhoid fever with blood-culture-confirmed non-typhoid bacterial infection (after excluding the contaminated specimens, including 127 from the TCV group and 151 from the Japanese encephalitis vaccine group) as there was no evidence that TCV or Japanese encephalitis vaccine was associated with these infections.

In typhoid-endemic countries, blood cultures are normally done in individuals with suspected typhoid rather than in all patients with fever.^26^ The selection for testing might introduce a collider bias as the testing is likely to be a collider of vaccination and infection. In our study, we further tested whether selecting TND participants based on clinical diagnosis introduces bias to the vaccine effectiveness estimate by repeating the TND analysis among the population with a clinical diagnosis of typhoid in the 75 TCV clusters. All analyses were performed in R version 4.2.2.

### Role of the funding source

The funder of the study had no role in study design, data collection, data analysis, data interpretation, or writing of the report.

## Results

**Figure 2:**
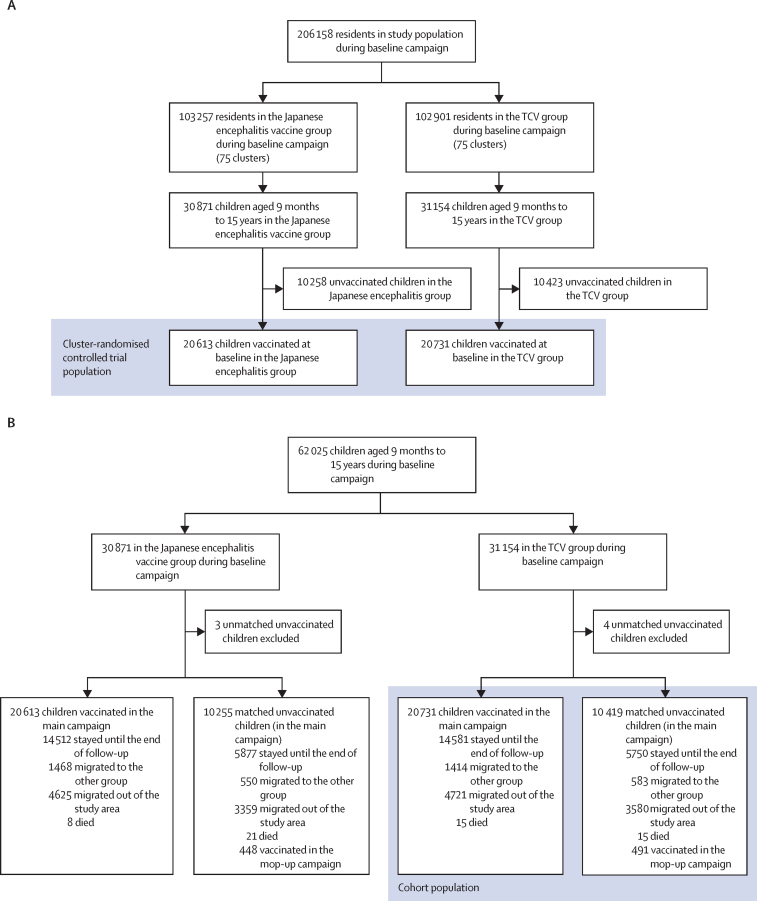

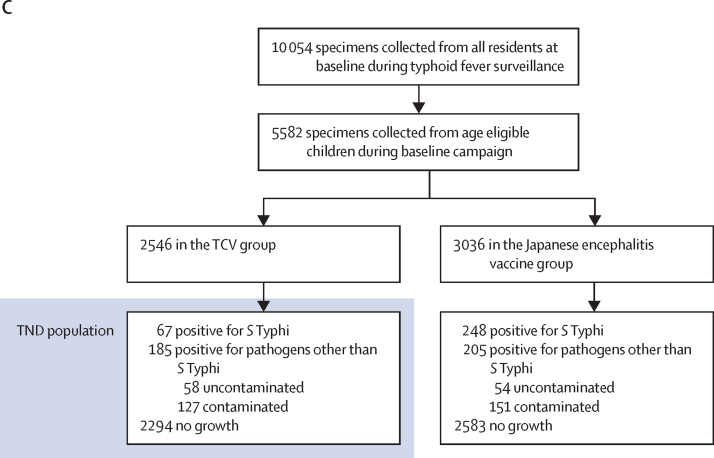
Consort diagrams for primary analysis in age-eligible children at baseline vaccination campaign (A) Cluster-randomised controlled trial. (B)Cohort design. (C) TND. *S* Typhi=*Salmonella enterica* serotype Typhi. TCV=typhoid conjugate vaccine. TND=test-negative case–control study design.

Between April 15 and May 15, 2018, 41 344 of 62 025 age-eligible children in the study area received the TCV or Japanese encephalitis vaccine during the baseline vaccination campaign (figure 2). Among the age-eligible children, median age was 8·2 years (IQR 4·4–12·2), 30 731 (50%) were female, and 31 294 (50%) were male. Imbalances were seen in baseline characteristics, household hygiene factors, and frequencies of visits to our surveillance hospitals with fever between vaccinated and unvaccinated children within both the TCV and Japanese encephalitis vaccine clusters (appendix 1 p 11). In the 62 025 age-eligible children, 5582 blood culture specimens were collected by passive surveillance, including 2546 specimens from the 75 TCV clusters (figure 2). Children with a positive blood culture were more likely to be unvaccinated compared with children who tested negative for *S* Typhi (appendix 1 p 14).

Variation was observed for vaccine protective effects estimated by different study designs (Table 1, Table 2). In the cluster-randomised controlled trial analysis, the incidence of typhoid fever was 621 per 100 000 person-years (95% CI 538–713) for recipients of Japanese encephalitis vaccine and 67 per 100 000 person-years (42–102) for recipients of TCV (table 1). The estimated vaccine effectiveness for TCV was 89% (95% CI 81–93). In the cohort analysis among the 75 TCV clusters, typhoid incidence was 303 per 100 000 person-years (95% CI 218–409) among non-vaccinees and 67 per 100 000 person-years (42–102) among recipients of TCV, resulting in a vaccine effectiveness of 79% (95% CI 70–86; table 1). Compared with the cohort analysis, the TND provided a more similar vaccine effectiveness estimate to that in the cluster-randomised controlled trial: the vaccine effectiveness was 88% (95% CI 79–93) when pan-negatives were used as test-negative controls, 90% (75–96) when culture-positive specimens for pathogens other than *S* Typhi with contaminants excluded were used as test-negative controls, and 88% (76–94) when culture-positive specimens for pathogens other than *S* Typhi with contaminants included were used as test-negative controls (table 2). For the traditional case–control design, the median of the vaccine effectiveness point estimate was 70% (IQR 61–77) by 1000 simulations, which was lower than the vaccine effectiveness estimate from the cluster-randomised controlled trial.

**Table 1:** Vaccine effectiveness against blood-culture-confirmed typhoid fever estimated by cluster-randomised controlled trial and cohort study

	Recipients of Japanese encephalitis vaccine or non-vaccinees*	**Recipients of TCV (n=20 731)**	**Adjusted IRR (95% CI)**†	**Vaccine effectivness (95% CI)**†	**p value**
**Cluster-randomised controlled trial (150 clusters)**					
Blood-culture-confirmed typhoid fever, n/person-years of follow-up	202/32 518	22/32 687	0·11 (0·06–0·19)	89% (81–93)	<0·0001
Incidence per 100 000 person-years (95% CI)	621 (538–713)	67 (42–102)	..	..	..
**Cohort study (75 TCV clusters)**					
Blood-culture-confirmed typhoid fever, n/person-years of follow-up	42/13 870	22/32 687	0·22 (0·15–0·31)	79% (70–86)	<0·0001
Incidence per 100 000 person-years (95% CI)	303 (218–409)	67 (42–102)	..	..	..

IRR=incidence rate ratio. TCV=typhoid conjugate vaccine.

* Recipients of Japanese encephalitis vaccine in the cluster-randomised controlled trial (n=20 613); non-vaccinees in the cohort study (n=10 419).

† IRR was adjusted for the stratifying variables for randomisation, including geographical ward, distance to study clinics (below or above median), number of eligible children at baseline, and age, sex, toilet type in the house, drinking water source, treatment of drinking water, handwashing before meals, and handwashing after defecation.

**Table 2:** Vaccine effectiveness against blood culture-confirmed typhoid fever estimated by test-negative case–control study design analyses

	**Test negatives**	**Test positives**	Adjusted OR (95% CI)*	Vaccine effectiveness (95% CI)*	**p value**
**Test negatives: positive for pathogens other than *S* Typhi excluding 127 contaminants**					
Non-vaccinees	10/58 (17%)	45/67 (67%)	0·10 (0·04–0·25)	90% (75–96)	<0·0001
Recipients of TCV	48/58 (83%)	22/67 (33%)	..	..	..
**Test negatives: positive for pathogens other than *S* Typhi including 127 contaminants**					
Non-vaccinees	39/185 (21%)	45/67 (67%)	0·12 (0·06–0·24)	88% (76–94)	<0·0001
Recipients of TCV	146/185 (79%)	22/67 (33%)	..	..	..
**Test negatives with no growth**					
Non-vaccinees	456/2294 (20%)	45/67 (67%)	0·12 (0·07–0·21)	88% (79–93)	<0·0001
Recipients of TCV	1838/2294 (80%)	22/67 (33%)	..	..	..

OR=odds ratio. *S* Typhi=*Salmonella enterica* serotype Typhi. TCV=typhoid conjugate vaccine.

* OR was adjusted for age, sex, toilet type in the house, drinking water source, treatment of drinking water, handwashing before meals, handwashing after defecation, distance to study clinics (numerical), and matched calendar month of fever start date.

In the Japanese encephalitis vaccine clusters, the incidence of typhoid fever among non-vaccinees was 301 per 100 000 person-years (95% CI 217–407), which was similar to that among non-vaccinees from the TCV clusters (Table 1, Table 3). In the cohort NCE analysis, the incidence rate ratio (IRR) for typhoid fever in recipients of Japanese encephalitis vaccine compared with non-vaccinees was 1·98 (95% CI 1·56–2·52), suggesting an increased risk of typhoid fever with Japanese encephalitis vaccination relative to those not receiving the vaccine. An increased risk associated with Japanese encephalitis vaccination was also seen in the traditional case–control NCE analyses, with a median adjusted odds ratio by 1000 simulations of 1·97 (IQR 1·72–2·22). In the TND NCE analysis, no such association was observed (table 4).

**Table 3:** The associations between Japanese encephalitis vaccination and blood-culture-confirmed typhoid fever by cohort analysis (75 Japanese encephalitis vaccine clusters, negative control exposure)

	**Non-vaccinees (n=10 255)**	**Recipients of Japanese encephalitis vaccine (n=20 613)**	Adjusted IRR (95% CI)*	**p value**
Blood-culture-confirmed typhoid fever, n/person-years of follow-up	42/13 955	202/32 518	1·98 (1·56–2·52)	<0·0001
Incidence per 100 000 person-years (95% CI)	301 (217–407)	621 (538–713)	..	..

IRR=incidence rate ratio.

* IRR was adjusted for the stratifying variables for randomisation, including geographical ward, distance to study clinics (below or above median), number of eligible children at baseline, and age, sex, toilet type in the house, drinking water source, treatment of drinking water, handwashing before meals, and handwashing after defecation.

**Table 4:** The associations between Japanese encephalitis vaccination and blood-culture-confirmed typhoid fever by test-negative case–control study design (75 Japanese encephalitis vaccine clusters)

	**Test negatives**	**Test positives**	Adjusted OR (95% CI)*	**p value**
**Test negatives: positive for pathogens other than *S* Typhi excluding 151 contaminants**				
Non-vaccinees	10/54 (19%)	46/248 (19%)	0·84 (0·37–1·92)	0·68
Recipients of Japanese encephalitis vaccine	44/54 (81%)	202/248 (81%)	..	..
**Test negatives: positive for positive for pathogens other than *S* Typhi including 151 contaminants**				
Non-vaccinees	45/205 (22%)	46/248 (19%)	1·15 (0·71–1·86)	0·56
Recipients of Japanese encephalitis vaccine	160/205 (78%)	202/248 (81%)	..	..
**Test negatives with no growth**				
Non-vaccinees	483/2583 (19%)	46/248 (19%)	1·04 (0·74–1·46)	0·83
Recipients of Japanese encephalitis vaccine	2100/2583 (81%)	202/248 (81%)	..	..

OR=odds ratio. *S* Typhi=*Salmonella enterica* serotype Typhi.

* OR was adjusted for age, sex, toilet type in the house, drinking water source, treatment of drinking water, handwashing before meals, handwashing after defecation, distance to study clinics (numerical), and matched calendar month of fever start date.

In the NCO analysis, the incidence for other infections was 138 per 100 000 person-years (95% CI 101–185) among recipients of Japanese encephalitis vaccine and 144 per 100 000 person-years (106–191) among recipients of TCV (table 5). There was no significant difference between the two groups, with an adjusted IRR of 1·08 (95% CI 0·70–1·69) in the cluster-randomised controlled trial analysis. In the cohort analysis, there was a 2–3-times increased risk of other infections among both recipients of Japanese encephalitis vaccine and recipients of TCV compared with non-vaccinees from their corresponding groups (table 5). Similar to the NCE analysis, these associations were not observed in the TND analyses, with an adjusted OR of 1·26 (95% CI 0·63–2·53) between test negatives (specimens with no growth) and test positives (positive for pathogens other than *S* Typhi after excluding contaminants) in the TCV clusters and 0·99 (0·49–2·01) in the Japanese encephalitis vaccine clusters (table 6).

**Table 5:** Associations between TCV or Japanese encephalitis vaccination and the risk of infections by pathogens other than *Salmonella enterica* serotype Typhi in the cluster randomised controlled trial and cohort studies

	Recipients of Japanese encephalitis vaccine or non-vaccinees*	**Recipients of TCV or Japanese encephalitis vaccine**†	**Adjusted IRR (95% CI)**‡	**p value**
**Cluster-randomised controlled trial (150 clusters)**				
Blood-culture-confirmed typhoid fever, n/person-years of follow-up	45/32 518	47/32 687	1·08 (0·70–1·69)	0·72
Incidence per 100 000 person-years (95% CI)	138 (101–185)	144 (106–191)	..	..
**Cohort study (75 TCV clusters)**				
Blood-culture-confirmed typhoid fever, n/person-years of follow-up	6/13 870	47/32 687	2·88 (1·58–5·26)	0·0006
Incidence per 100 000 person-years (95% CI)	43 (16–94)	144 (106–191)	..	..
**Cohort study (75 Japanese encephalitis vaccine clusters)**				
Blood-culture-confirmed typhoid fever, n/person-years of follow-up	7/13 955	45/32 518	2·45 (1·38–4·35)	0·0022
Incidence per 100 000 person-years (95% CI)	50 (20–103)	138 (101–185)	..	..

IRR=incidence rate ratio. TCV=typhoid conjugate vaccine.

* Recipients of Japanese encephalitis vaccine in the cluster-randomised controlled trial (n=20 613); non-vaccinees in the cohort study in TCV clusters (n=10 419); non-vaccinees in the cohort study in Japanese encephalitis vaccine clusters (n=10 255).

† Recipients of TCV in the cluster-randomised controlled trial (n=20 731); recipients of TCV in the cohort study in TCV clusters (n=20 731); recipients of Japanese encephalitis vaccine in the cohort study in Japanese encephalitis vaccine clusters (n=20 613).

‡ IRR was adjusted for the stratifying variables for randomisation, including geographical ward, distance to study clinics (below or above median), number of eligible children at baseline, and age, sex, toilet type in the house, drinking water source, treatment of drinking water, handwashing before meals, and handwashing after defecation.

**Table 6:** Associations between TCV or Japanese encephalitis vaccination and the risk of infections by pathogens other than *S* Typhi in the TND analyses

		**Test negatives with no growth**	**Test positives**	Adjusted OR (95% CI)*	**p value**
**TND analysis in 75 TCV clusters**					
Test positives, positive for pathogens other than *S* Typhi excluding 127 contaminants					
	Non-vaccinees	456/2294 (20%)	10/58 (17%)	1·26 (0·63–2·53)	0·52
	Recipients of TCV	1838/2294 (80%)	48/58 (83%)	..	..
Test positives, positive for pathogens other than *S* Typhi including 127 contaminants					
	Non-vaccinees	456/2294 (20%)	39/185 (21%)	0·95 (0·66–1·38)	0·81
	Recipients of TCV	1838/2294 (80%)	146/185 (79%)	..	..
**TND analysis in 75 Japanese encephalitis vaccine clusters**					
Test positives, positive for pathogens other than *S* Typhi excluding 151 contaminants					
	Non-vaccinees	483/2583 (19%)	10/54 (19%)	0·99 (0·49–2·01)	0·99
	Recipients of Japanese encephalitis vaccine	2100/2583 (81%)	44/54 (81%)	..	..
Test positives, positive for pathogens other than *S* Typhi including 151 contaminants					
	Non-vaccinees	483/2583 (19%)	45/205 (22%)	0·84 (0·59–1·19)	0·33
	Recipients of Japanese encephalitis vaccine	2100/2583 (81%)	160/205 (78%)	..	..

OR=odds ratio. *S* Typhi=*Salmonella enterica* serotype Typhi. TCV=typhoid conjugate vaccine. TND=test-negative case–control study design.

* OR was adjusted for age, sex, toilet type in the house, drinking water source, treatment of drinking water, handwashing before meals, handwashing after defecation, distance to study clinics (numerical), and matched calendar month of fever start date.

When we expanded the study population to include all age-eligible children at any of the four study vaccination campaigns (appendix 1 p 10), the results were consistent with our primary analysis (appendix 1 p 17). The vaccine effectiveness estimate by the TND was similar to the cluster-randomised controlled trial analysis (83% [95% CI 76–88]) regardless of the choice of test-negative controls: 91% (80–96) for specimens positive for non-*S* Typhi pathogens excluding contaminants; 85% (75–91) for specimens positive for non-*S* Typhi pathogens including contaminants; and 82% (74–88) for pan-negative specimens. However, the vaccine effectiveness estimate was lower in the cohort analysis (67% [56–76]). Similar to the primary analysis, the cohort NCE analysis in the Japanese encephalitis vaccine clusters showed a doubled increase in the risk of typhoid fever among recipients of Japanese encephalitis vaccine, which was not seen in the TND NCE analysis (appendix 1 p 19). Similar patterns were seen in the NCO sensitivity analyses to the primary analyses (appendix 1 p 21).

When restricting the TND analysis in children with a clinical typhoid diagnosis, the results were similar to the primary TND analysis (appendix 1 p 23). The vaccine effectiveness estimate was 90% (95% CI 82–94) when using pan-negatives as test-negative controls and 96% (87–99) when using specimens positive for non-*S* Typhi pathogens (excluding contaminants) as test-negative controls. Similarly, no association between Japanese encephalitis vaccination and the risk of typhoid fever was observed in the NCE analysis (appendix 1 p 23).

## Discussion

Our analysis showed that the TND produced estimates of vaccine effectiveness for TCV that are very similar to the gold-standard estimates of vaccine effectiveness arising from a cluster-randomised controlled trial. Using NCE and NCO analyses, we found no association in the TND between Japanese encephalitis vaccination and typhoid fever or between TCV or Japanese encephalitis vaccination and non-*S* Typhi infections. However, cohort and traditional case–control analyses tended to underestimate vaccine effectiveness, possibly due to under-detection of cases in the unvaccinated group. This finding is consistent with our NCE results, which showed a doubled risk of typhoid fever among recipients of Japanese encephalitis vaccine compared with non-vaccinees in the cohort analysis and the traditional case–control analysis (which would be an association with no biologically feasible explanation). Additionally, NCO analysis showing a 2–3-times higher risk of non-*S* Typhi infections among recipients of TCV and Japanese encephalitis vaccine further showed the bias introduced by the cohort design.

Given the sensitivity of blood culture for typhoid diagnosis is only 59%,^27^ misclassification bias can influence vaccine effectiveness estimates across all study designs. However, the TND is particularly susceptible to this bias because the accuracy of the classification of cases and controls relies on high sensitivity of blood-culture tests.^27^ When typhoid cases are misclassified as test-negative controls, it reduces the difference in odds of vaccination between test-positive cases and test-negative controls, leading to an underestimation of vaccine effectiveness. The effect of this bias depends on the proportion of false negatives among all test-negative controls. Liang and colleagues^12^ simulated TNDs using data from a TCV randomised controlled trial in Malawi and confirmed that blood-culture sensitivity was unlikely to affect vaccine effectiveness estimates when typhoid fever accounts for less than 10% of the total blood culture. Their findings are supported by our study in which the low blood-culture sensitivity has a minimal effect on vaccine effectiveness estimates by the TND in Bangladesh, where the proportion of typhoid fever was 3% in TCV clusters and 9% in Japanese encephalitis vaccine clusters.

Vaccine effectiveness estimates by the TND were consistent with those by the cluster-randomised controlled trials, although some differences were seen depending on the choice of test-negative controls. Owing to the substantially larger number of pan-negative specimens than test-positive specimens for other pathogens, the 95% CI for the vaccine effectiveness estimate by pan-negative controls was notably narrower. For future TNDs, we recommend reporting vaccine effectiveness estimates using both pan-negative specimens and those test-positive for other pathogens to monitor the effect of false negatives due to low blood-culture sensitivity.

Cohort studies for vaccine effectiveness estimation are often integrated into existing population-based investigations or focus on specific groups with more manageable follow-up.^28^, ^29^ Individuals opting for vaccination can differ in many ways from those who choose not to receive a vaccine, including demographic factors such as age, gender, socioeconomic status, and health-care-seeking behaviours.^30^, ^31^ Our study showed that the number of visits to our surveillance hospitals with fever differed between vaccinees and non-vaccinees in Japanese encephalitis vaccine clusters, and this difference is likely to explain the biased results in the cohort design. Although using an emulated target trial approach might mitigate bias in vaccine effectiveness estimates, unmeasured confounding factors, such as health-care-seeking behaviours, might still lead to an underestimation of vaccine effectiveness,^21^ as observed in our study. This point was further illustrated by our NCE and NCO analyses, in which no association between vaccination and risk of infection was expected, yet consistent 2–3-times increased risk of infection in vaccine recipients compared with non-vaccinees were found after adjustment. Interestingly, the results from the traditional case–control design using community controls showed similar results to the cohort analyses. A possible explanation is that the potentially different health-care-seeking behaviours between vaccinees and non-vaccinees can bias cohort studies and traditional case–control studies in the same manner.

TNDs have been widely used to monitor vaccine effectiveness, particularly during the COVID-19 pandemic.^32^, ^33^ Concerns have been raised regarding the potential collider bias in TND studies, in which participants are conditionally selected based on diagnostic testing. Testing was considered as a collider because both vaccination and infection can increase the likelihood of testing.^15^, ^34^, ^35^ When only tested participants are selected for a study, an artificial association between vaccination and disease can possibly be presented. In our study, testing of blood culture was offered to all participants with predefined symptoms that were not specific to typhoid (at least 2 days of fever or with an axillary temperature of at least 38°C), and all participants with a blood culture taken were recruited. The TND NCE analysis did not show an increased risk between the Japanese encephalitis vaccine and typhoid fever, suggesting that this recruitment approach is unlikely to introduce collider bias. In real-world TND studies, testing participants with non-specific symptoms can be challenging in countries with low resources, especially when the TND study is conducted retrospectively using hospital records or electronic registration datasets. In such situations, the selection of samples for testing cannot be controlled by study investigators. To further understand the effect of selection for blood culture in real settings, we restricted our TND analysis to participants with a clinical diagnosis of typhoid, and similar results were seen as in the main TND analyses. Because there was probably a link between vaccination and testing (ie, vaccinees had more fever visits and blood cultures taken), a possible explanation for not seeing a collider bias is that the clinical diagnosis of typhoid is not sensitive. These findings provide further reassurance regarding the validity of the TND in measuring vaccine effectiveness of TCVs following introduction because no obvious collider bias was detected. In future TND studies, we recommend incorporating NCE and NCO methods, when possible, to monitor sources of bias, ensuring an accurate interpretation of TND results.

One limitation of this study is that the cohort and TND analyses were done using data collected in a cluster-randomised controlled trial, in which study procedures are generally more comprehensive than real-world observational studies. Therefore, interpreting results from real-world observational studies should be approached cautiously, considering differences in study designs and settings. The representation of trial participants in our study was less of a concern because all age-eligible and healthy children in the study area without plans to move out within 1 month were invited to participate in the trial and the average vaccine coverage was 65%. In this cluster-randomised controlled trial, vaccination campaigns were conducted over a short period, and vaccination records were well documented. By contrast, real TND studies often collect these data retrospectively, leading to potential misclassification bias.^12^ In our study, NCE analysis was conducted in the Japanese encephalitis vaccine clusters rather than the TCV clusters, whereas NCE and the main analyses are done in the same population in real-world TNDs. This means we were unable to directly evaluate potential bias of vaccine effectiveness estimates by NCE analysis. Nonetheless, we believe that our results are still valid as all trial clusters were randomly assigned, and participants were masked to their study group. The consistency between NCO analyses in the TCV and Japanese encephalitis vaccine clusters further supports that the two groups of randomly assigned clusters are comparable and that our TND results are unlikely to be biased. Last, our study did not measure individual health-care-seeking behaviour directly so we could not adjust for that in our analysis. Although non-vaccinees had fewer visits to our surveillance hospitals with fever than vaccinees during follow-up, it is possible that the non-vaccinees were generally healthier than vaccinees, rather than having less propensity to seek health care when ill. However, based on the baseline characteristics of vaccinees and non-vaccinees (ie, similar socioeconomic and water–sanitation–hygiene factors), it is unlikely to be that the non-vaccinees were healthier. Further prospective studies in this population are needed to confirm the difference in health-care-seeking behaviours between the two populations.

In conclusion, our findings validate the TND as an efficient and reliable method to monitor vaccine effectiveness following vaccine introduction. We recommend the adoption of TND studies alongside bias identification approaches such as NCE and NCO to monitor vaccine effectiveness in low-resource settings.

### Contributors

### Equitable partnership declaration

### Data sharing

De-identified individual participant data including a data dictionary for each variable analysed in this report will be made available when the original trial is complete, upon requests directed to the corresponding author. Only after approval of a proposal can data be shared through a secure online platform. Approval of the proposal will be subject to scientific review by the institutional review board at the International Centre for Diarrhoeal Disease Research, Bangladesh. Sharing of data will also be subject to the published data access rules of the International Centre for Diarrhoeal Disease Research, Bangladesh. The requestor will need to sign a standard data access agreement required by the International Centre for Diarrhoeal Disease Research, Bangladesh.

## Declaration of interests

AJP is chair of the UK Department of Health and Social Care's Joint Committee on Vaccination; was a member of WHO's Strategic Advisory Group of Experts until 2022; and is chair of WHO's Technical Advisory Group on *Salmonella* vaccines. The University of Oxford has entered a partnership with AstraZeneca for the development of a COVID-19 vaccine; AJP and XL are contributors to the intellectual property licensed by Oxford University Innovation to AstraZeneca. FQ, VEP, and XL are members of the WHO Strategic Advisory Group of Experts typhoid working group. XL is a member of the WHO Technical Advisory Group on *Salmonella* vaccines and is an independent member of several data and safety monitoring boards and one trial steering committee. VEP was previously a member of the WHO Immunization and Vaccine related Implementation Research Advisory Committee. AJP receives grants from the Wellcome Trust, the Coalition for Epidemic Preparedness Innovations, Medical Research Council, National Institute for Health and Care Research, AstraZeneca, European Commission, and Serum Institute of India. VEP receives grants from Gavi, the Vaccine Alliance, US Centers for Disease Control and Prevention, National Institutes of Health/National Institute of Allergy and Infectious Diseases, and National Institute for Health and Care Research. All other authors declare no competing interests.

## Supplementary Materials

Supplementary appendix 1

Equitable partnership declaration
