# Supplementary material for: The validity of test-negative design for assessment of typhoid conjugate vaccine protection: comparison of estimates by different study designs using data from a cluster-randomised controlled trial
Source: Lancet Glob Health. 2025 Apr 16;13(6):e1122–31. doi: 10.1016/S2214-109X(25)00056-7 (PMC12095117; doi:10.1016/S2214-109X(25)00056-7)
Supplement: Supplementary appendix 1 [file mmc1.pdf]

THE LANCET
Global Health

Supplementary appendix 1

This appendix formed part of the original submission and has been peer reviewed. We post it as supplied by the authors.

Supplement to: Feng S, Zhang Y, Khanam F, et al. The validity of test-negative design for assessment of typhoid conjugate vaccine protection: comparison of estimates by different study designs using data from a cluster-randomised controlled trial. *Lancet Glob Health* 2025; published online April 16. https://doi.org/10.1016/S2214-109X(25)00056-7.

The validity of test-negative design for assessment of typhoid conjugate vaccine protection: comparison of estimates by different study designs using data from a cluster randomised controlled clinical trial

Supplementary Materials

Table of contents

Statistical Methods 3
Supplementary Figure 1. Timeline of the study 8
Supplementary Figure 2. Time trends of blood culture results 9
Supplementary Figure 3. Consort diagram for sensitivity analysis in all age-eligible children at any of the vaccination campaigns during the study 10
Supplementary Table 1. Demographic characteristics by cohort analysis 11
Supplementary Table 2. Demographic characteristics by the test-negative study design in TCV clusters 14
Supplementary Table 3. Vaccine protection against blood-culture confirmed typhoid fever in the sensitivity analyses among all age-eligible children at any of the four vaccination campaigns during the study period$^{\pm}$ 17
Supplementary Table 4. The associations between JE vaccination and blood culture-confirmed typhoid fever in the sensitivity analyses among all age-eligible children at any of the four vaccination campaigns during the study period$^{\pm}$ 19
Supplementary Table 5. Associations between TCV or JE vaccination and the risk of infections by pathogens other than S.Typhi in the sensitivity analyses among all age-eligible children at any of the four vaccination campaigns during the study period$^{\pm}$ 21
Supplementary Table 6. Sensitivity TND analysis in specimens with a clinical diagnosis of typhoid among all age-eligible children at any of the four vaccination campaigns during the study period $^{\pm}$ 23
References 25

Statistical Methods

CRCT Analysis

In the CRCT analysis of all 150 clusters, we included all the children who received one dose of TCV or JE as our aim is to estimate the direct protection by TCV to allow for the comparison with VE estimates from the TND and the cohort study design. The follow-up time was calculated between the date of vaccination and the earliest date among the date of death, the end of surveillance, or the date of moving out of their initial vaccination arm, including moving to clusters of the other arm or outside the study area. The VE against blood-culture-confirmed typhoid fever was calculated as (1 - adjusted incidence rate ratio (IRR_{adj})) ×100% and the IRR between TCV and JE arm was estimated by the mixed-effects Poisson regression model with the outcome being the number of blood-culture-confirmed typhoid fever episodes for each participant. The cluster variable was included as a random effect in the model and fixed effects include randomisation stratification variables at the cluster level (geographical ward, distance to study clinics, number of eligible children at baseline), demographic factors (age and sex), and household hygiene factors (toilet type in the house, drinking water source, treatment of drinking water, and handwashing practices before meals and after defecation).The inverse probability of censoring weighting (IPCW) was used to estimate VE to control the potential impact of censoring. For IPCW, we first fitted a logistic regression model with censoring as the outcome with the same set of covariates in the Poisson regression as predictors. We then used predicted probabilities from this model to calculate inverse probability weights, which were applied to each participant in the Poisson regression model.

Cohort Analysis

For the cohort analysis, we emulated a hypothetical target trial among residents in the 75 TCV clusters.[1-3] In the primary analysis, we included all children who met the eligibility criteria at the baseline vaccination campaign (Figure 2). These children were grouped into vaccinees and non-vaccinees based on their vaccination status at the baseline campaign. The follow-up time for vaccinees in the cohort analysis aligned with that of the CRCT analysis. For non-vaccinees, we employed a campaign-based matching approach to assign time zero, ensuring comparability with vaccinees. For each non-vaccinee, we identified a pool of matched vaccinees from the baseline campaign based on age (in years), gender, and geographical ward. From this pool, we randomly selected one vaccinated child whose date of vaccination was assigned as time zero for the non-vaccinee. We did not match on all potential confounders, as it resulted in over-matching, and in some cases, non-vaccinees had no matched vaccinated children if all confounders were included. However, these confounders were adjusted in the regression model. The follow-up time for non-vaccinees was censored in the same way as vaccinees. Unvaccinated children who later received a TCV at a catch-up vaccination campaign were also censored at the time of vaccination. We estimated the IRR by comparing the incidence rate of blood-culture-confirmed typhoid fever among vaccinated children to that of unvaccinated children using the same Poisson regression model as in the CRCT analysis. The IPCW was also used in the cohort analysis. In the sensitivity analysis, participants who migrated in or became eligible after the baseline campaign were included, and the same analysis method as the primary analysis was used.

TND analysis

In the TND analysis, we adopted a specimen-based approach, where units of analysis were blood culture specimens rather than participants, without censoring for typhoid positivity to

account for the multiple fever visits during the follow-up period.[4] In the primary analysis, all the specimens from age-eligible children at the baseline vaccination campaign in the 75 TCV clusters were included. The sensitivity analysis extended to specimens from all age-eligible children at any of the vaccination campaigns. The specimens from children who moved into TCV clusters from JE clusters were excluded as they were also censored in the above CRCT and cohort analyses at the time of moving. A total of 278 specimens with cultures positive for other pathogens were identified as contaminants, predominantly consisting of coagulase-negative *Staphylococci* and *Micrococcus* species. Since these contaminant bacteria can create a competitive environment in the culture plate and we cannot confirm if they were true test-negatives, we conducted two analyses by including and excluding them as test-negative controls. A conditional logistic regression model matching by calendar month to control seasonality (Supplementary Figure 2, page 3) was used to estimate the odds ratio (OR) of vaccination for test-positive cases versus test-negative controls adjusting for the same set of covariates in the CRCT and cohort analyses, and the VE was estimated by $(1–OR_{adj}) \times 100\%$.[5] Children were classified as vaccinated if they had received a TCV before the fever visit.

Matched case-control analysis

To validate the theoretical advantage of the TND over traditional matched case-control design in reducing biases, we simulated 1000 matched case-control studies. A real matched case-control study normally matches a certain number of controls with each case and retrospectively collects the vaccination data from the selected controls. Since we collected the data for all the controls prospectively and have multiple controls available to match with each case, we decided to run 1000 simulated studies to account for the impact of random sampling for the matching. In each of the simulated studies, we randomly selected three community controls for each blood-culture-confirmed typhoid case, matching by age (+/- one year) and

geographical location (in the same cluster). The VE was estimated by (1–OR_{adj}) ×100%, and the OR_{adj} was modelled by the conditional logistic regression model by matching pair, adjusting for the same set of covariates in the TND.

NCE and NCO analysis

We further utilised a negative control exposure (NCE) and a negative control outcome (NCO) approaches to assess potential biases in the CRCT (NCO only), cohort, traditional matched case-control, and TND analysis.[6-8] Negative control approaches have been increasingly used in epidemiology studies, including those assessing vaccine effectiveness, to identify confounding, selection, and measurement biases.[9] An NCE method tests an exposure that is susceptible to the same sources of bias as the primary exposure but not causally affects the outcome. Similarly, an NCO method uses an outcome that is not plausibly affected by the exposure of interest. It is expected that there will be no association between the NCE and the outcome of interest and between the exposure of interest and the NCO, if the study design is not subjected to bias. In our NCE analysis, the above cohort and TND analyses were repeated in the 75 JE clusters taking JE vaccination as the negative exposure as the JE vaccine provides no protection against typhoid fever. For the NCO analysis, we replaced the outcome of typhoid fever with blood-culture-confirmed non-typhoid bacterial infection (after excluding the contaminated specimens) as there was no evidence that TCV or JE associated with these infections, and repeated the cohort and TND analyses in the TCV and JE clusters, separately, assuming that TCV and JE vaccines do not protect against other bacterial infections.

Assessment of collider bias

A "collider" is defined as one variable that is independently influenced by both an exposure and an outcome. A collider bias can occur when a collider is inappropriately controlled. In the TND analysis to estimate VE, collider bias occurs when the study population is selected based

on diagnostic testing,[10] where the diagnostic testing is likely to be a collider of vaccination and infection. This bias can create an artificial vaccine protection even when no true protective effect exists. In our study, we would like to test if selecting TND participants based on fever visits introduces the collider bias on the VE estimate. If the TND is affected by the collider bias, an artificial association would be observed in the NCE analysis. In typhoid-endemic countries, blood cultures are normally performed in suspected typhoid cases rather than in all fever patients due to limited resources.[11] TND studies to monitor the TCV VE have been conducted based on this selection for blood cultures. To further evaluate the impact of this selection, we restricted our TND analysis to participants with a clinical diagnosis of suspected typhoid by healthcare professionals prior to obtaining blood culture results.

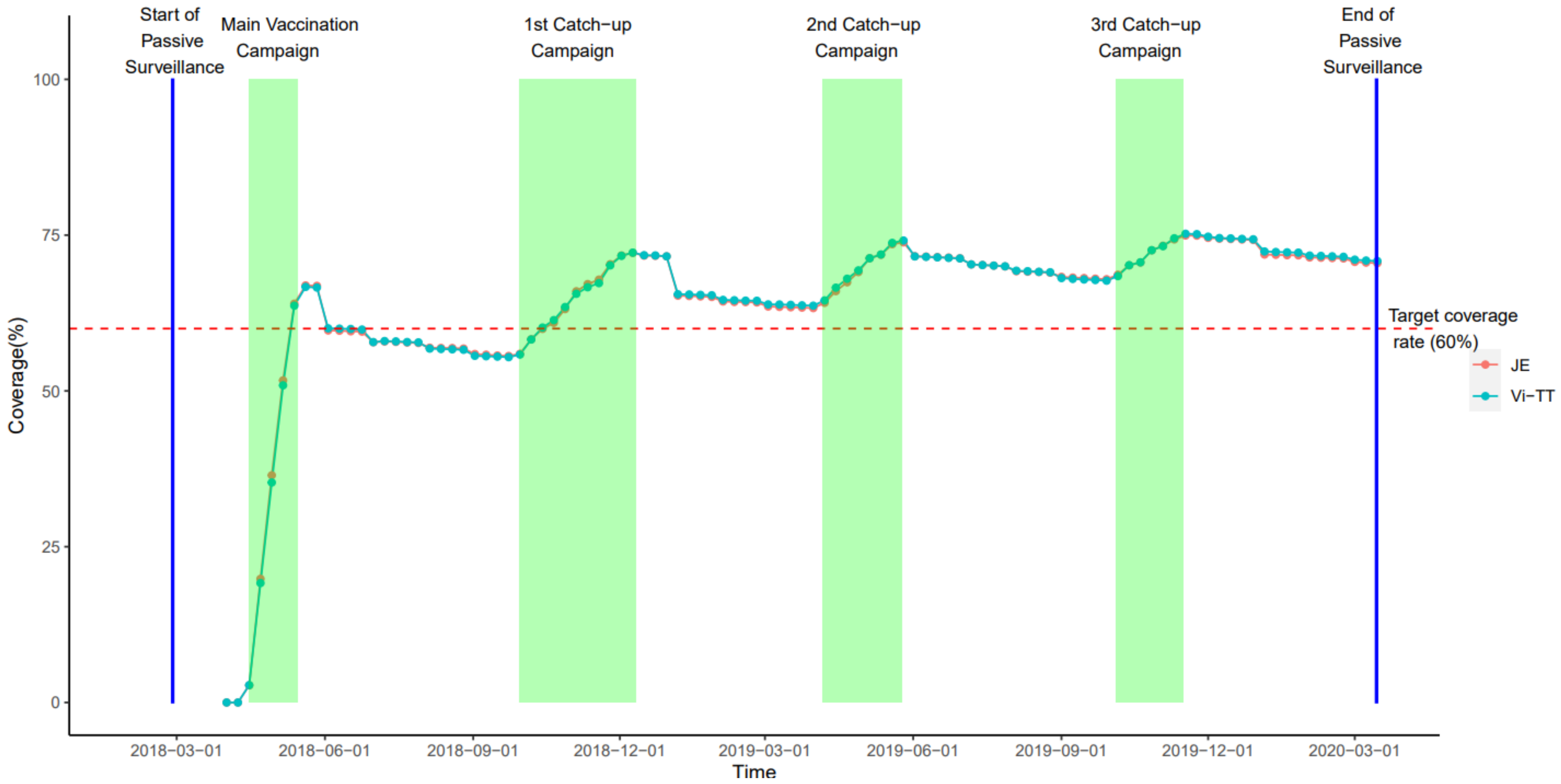

Supplementary Figure 1. Timeline of the study

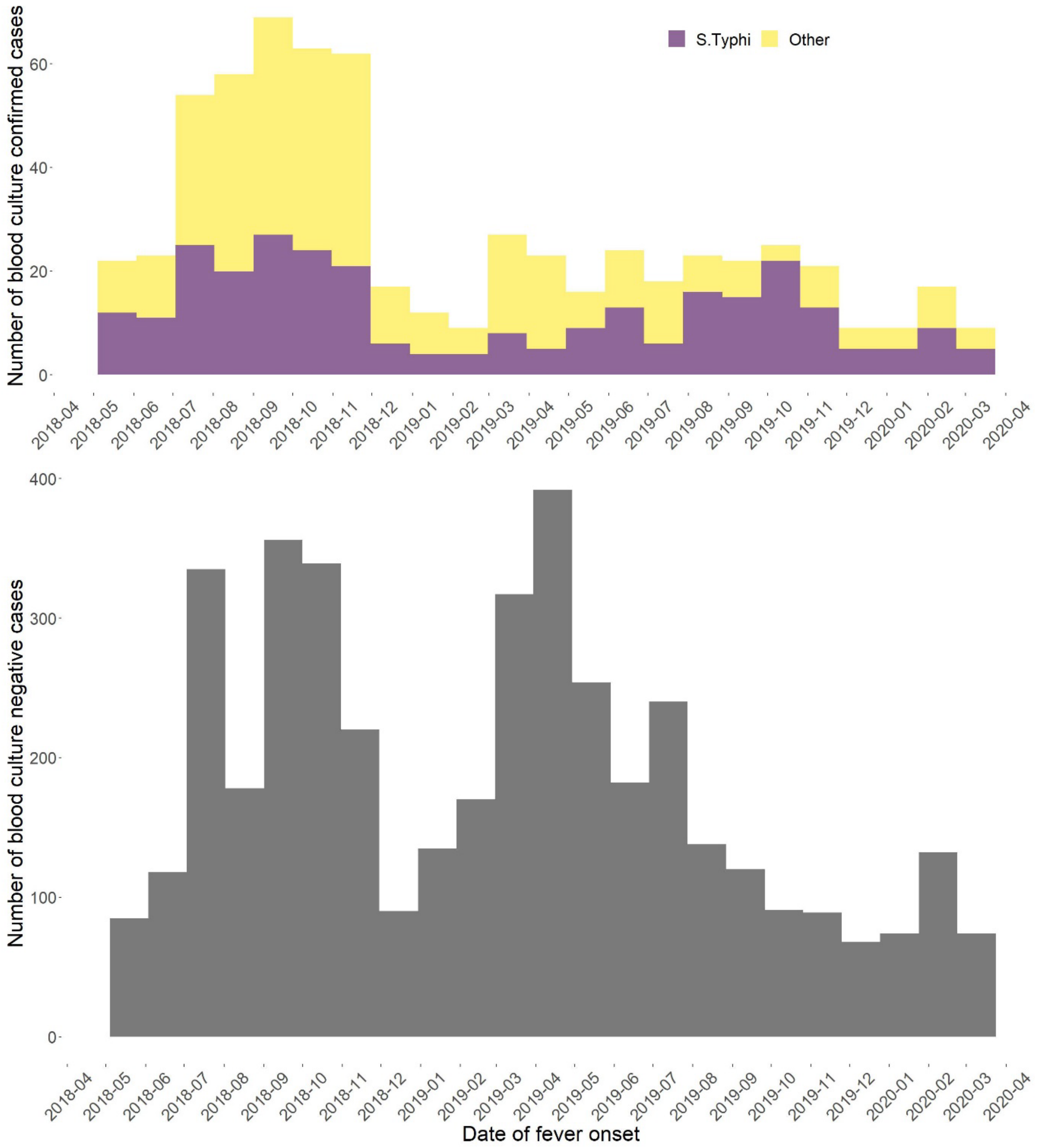

Supplementary Figure 2. Time trends of blood culture results

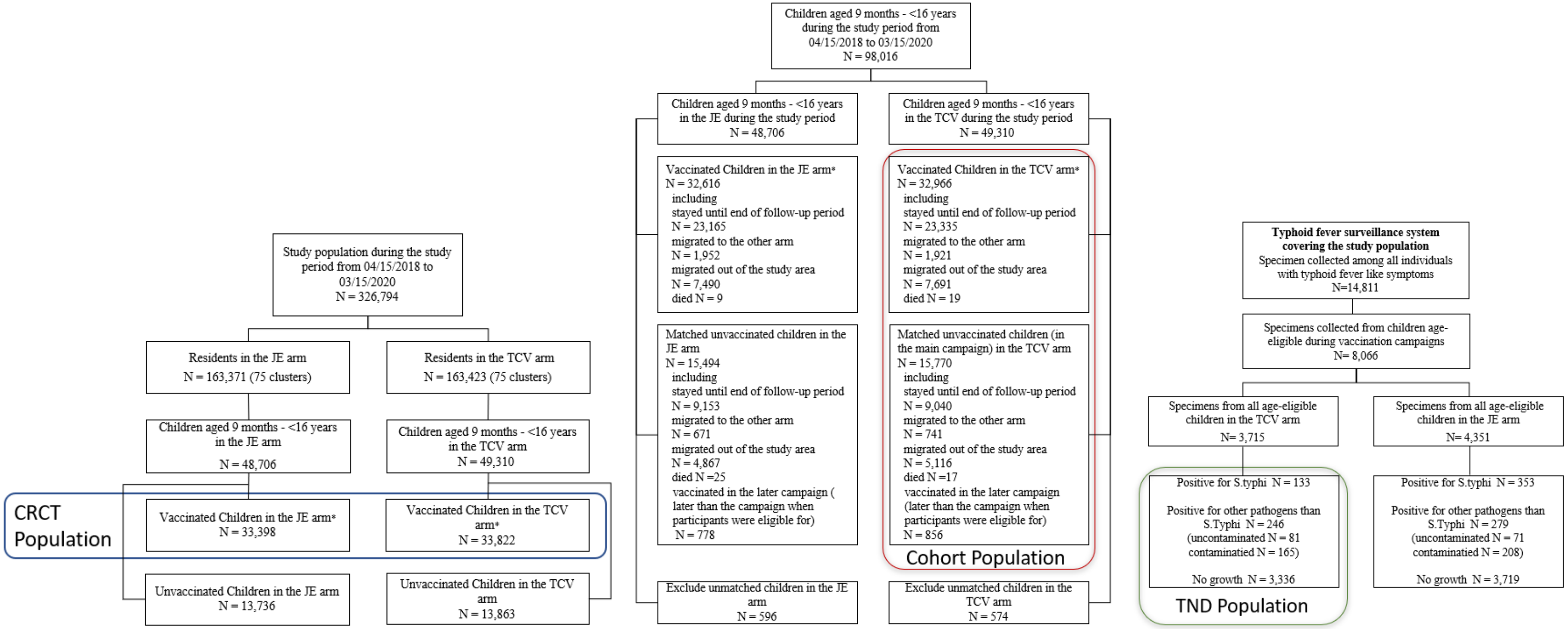

Supplementary Figure 3. Consort diagram for sensitivity analysis in all age-eligible children at any of the vaccination campaigns during the study

* Vaccinated children in the TCV arm in CRCT population (N = 33,822) include vaccinated children in the cohort analysis (N = 32,966) and those unvaccinated at day zero but vaccinated at a later campaign (N = 856). Vaccinated children in the JE arm in the CRCT population (N = 33,398) include vaccinated children in the cohort analysis (N = 32,616) and those unvaccinated at day zero but vaccinated at a later campaign (N = 778), and 4 children who were unvaccinated at day zero and failed to match with a vaccinated child in the cohort analysis.

Supplementary Table 1. Demographic characteristics by cohort analysis

	TCV clusters		JE clusters	
	**Vaccinated**	**Unvaccinated**	**Vaccinated**	**Unvaccinated**
**All eligible children aged 9 months to 15 years**				
	N=20731	N=10419	N=20613	N=10255
**Mean/Median duration of follow-up**				
**Age, years** †	8.1 (4.2) [n=20731]	8.8 (4.9) [n=10419]	8.0 (4.2) [n=20613]	8.8 (4.9) [n=10255]
**Sex**				
Female	10563 (51%)	4917 (47%)	10333 (50%)	4913 (48%)
Male	10168 (49%)	5502 (53%)	10280 (50%)	5342 (52%)
**Ward of residence**				
2	8395 (41%)	4033 (39%)	7713 (37%)	3697 (36%)
3	5193 (25%)	2799 (27%)	4584 (22%)	2473 (24%)
5	7143 (35%)	3587 (34%)	8316 (40%)	4085 (40%)
**Distance to study site, metres**†	417.9 (190.9) [n=20731]	419.7 (196.4) [n=10419]	423.6 (208.5) [n=20613]	415.4 (205.4) [n=10255]
**Attends school**				
Yes	13865 (67%)	5960 (57%)	13660 (66%)	5983 (58%)
No	2006 (10%)	1832 (18%)	2036 (10%)	1783 (17%)
Unknown	4860 (23%)	2627 (25%)	4917 (24%)	2489 (24%)
**Religion**				

Muslim	20477 (99%)	10279 (99%)	20445 (99%)	10154 (99%)
Others [1]	254 (1%)	140 (1%)	168 (1%)	101 (1%)
**Type of toilet**				
Flush toilet	859 (4%)	626 (6%)	789 (4%)	595 (6%)
Others [2]	19872 (96%)	9793 (94%)	19824 (96%)	9660 (94%)
**Source of drinking water**				
Own sources	5070 (25%)	2992 (29%)	5634 (27%)	3247 (32%)
Others [3]	15661 (76%)	7427 (71%)	14978 (73%)	7008 (68%)
**Type of drinking water**				
Treated [4]	6492 (31%)	3224 (31%)	5965 (29%)	2979 (29%)
Untreated	14239 (69%)	7195 (69%)	14643 (71%)	7275 (71%)
Unknown	0 (0%)	0 (0%)	5 (0%)	1 (0%)
**Wash hand with soap before meal**				
Yes	14769 (71%)	7500 (72%)	13937 (68%)	7049 (69%)
No	5962 (29%)	2919 (28%)	6676 (32%)	3206 (31%)
**Wash hand with soap after defecation**				
Yes	20157 (97%)	10084 (97%)	20026 (97%)	9971 (97%)
No	574 (3%)	335 (3%)	587 (3%)	284 (3%)
**Did the household have saving? ***				

Yes	4416 (21%)	2197 (21%)	3835 (19%)	2020 (20%)
No	16059 (78%)	8039 (77%)	16450 (80%)	8036 (78%)
Unknown	256 (1%)	183 (2%)	328 (2%)	199 (2%)
**Monthly saving (for who have saving) *†**	16.6 (18.0) [n=4416]	17.4 (16.1) [n=2197]	17.8 (16.4) [n=3835]	19.8 (20.0) [n=2020]
**Household monthly approximate expenditure ‡**	153.6 (124.8-201.6) [n=20726]	153.6 (120.0-206.4) [n=10419]	148.8 (115.2-192.0) [n=20567]	148.8 (115.2-201.6) [n=10219]
**Average number of fever visits within study period (cluster-level), days‡**	0.11 (0.08-0.15) [n=20731]	0.05 (0.03-0.07) [n=10419]	0.14 (0.11-0.17) [n=20613]	0.05 (0.03-0.08) [n=10255]
**Number of fever visits within study period**				
0	18689 (90%)	9906 (95%)	18141 (88%)	9719 (95%)
>=1	2042 (10%)	513 (5%)	2471 (12%)	536 (5%)

[1] refers to Hindu, Christian, Buddhist, or other religions;
[2] refers to toilet without flush, no toilet, or open space;
[3] refers to communal tap, communal tube well, communal hand pump, water vendor, stored in reservoir, pond/canal/river, shared tap/tube well/well in household, or others;
[4] refers to boiled, filtered, or chemicals treated;
* Children who were from the same households were counted more than once;
† mean(sd)
‡ median (IQR)
1.00 Bangladeshi Taka = 0.0096 US Dollar (the rate on 5th Jan 2023)

Supplementary Table 2. Demographic characteristics by the test-negative study design in TCV clusters

	Test positives	Test negatives (Positive for other pathogens, uncontaminated)	Test negatives (Positive for other pathogens, contaminated)	Test negatives (PAN negatives)
**Total**				
	N=67	N=58	N=127	N=2294
**Age , years**†	6.5 (3.8) [n=67]	7.8 (4.9) [n=58]	6.5 (4.7) [n=127]	7.1 (4.6) [n=2294]
**Sex**				
Female	30 (44.8%)	27 (46.6%)	60 (47.2%)	1027 (44.8%)
Male	37 (55.2%)	31 (53.4%)	67 (52.8%)	1267 (55.2%)
**Geographical Ward**				
2	22 (32.8%)	15 (25.9%)	34 (26.8%)	705 (30.7%)
3	12 (17.9%)	15 (25.9%)	37 (29.1%)	612 (26.7%)
5	33 (49.3%)	28 (48.3%)	56 (44.1%)	977 (42.6%)
**Distance to study site, metres**†	458.5 (224.1) [n=67]	463.0 (205.4) [n=58]	385.7 (190.5) [n=127]	410.9 (201.3) [n=2294]
**Attends school**				
Yes	36 (53.7%)	34 (58.6%)	53 (41.7%)	1128 (49.2%)
No	6 (9.0%)	5 (8.6%)	13 (10.2%)	205 (8.9%)
Unknown	25 (37.3%)	19 (32.8%)	61 (48.0%)	961 (41.9%)
**Religion**				

Muslim	67 (100%)	58 (100%)	125 (98.4%)	2274 (99.1%)
Others [1]	0 (0%)	0 (0%)	2 (1.6%)	20 (0.9%)
**Type of toilet**				
Flush toilet	2 (3.0%)	3 (5.2%)	6 (4.7%)	83 (3.6%)
Others [2]	65 (97.0%)	55 (94.8%)	121 (95.3%)	2211 (96.4%)
**Source of drinking water**				
Own sources	18 (26.9%)	13 (22.4%)	34 (26.8%)	530 (23.1%)
Others [3]	49 (73.1%)	45 (77.6%)	93 (73.2%)	1764 (76.9%)
**Type of drinking water**				
Treated [4]	49 (73.1%)	46 (79.3%)	89 (70.1%)	1639 (71.4%)
Untreated	18 (26.9%)	12 (20.7%)	38 (29.9%)	655 (28.6%)
**Wash hand with soap before meal**				
Yes	46 (68.7%)	37 (63.8%)	91 (71.7%)	1661 (72.4%)
No	21 (31.3%)	21 (36.2%)	36 (28.3%)	633 (27.6%)
**Wash hand with soap after defecation**				
Yes	66 (98.5%)	58 (100%)	124 (97.6%)	2252 (98.2%)
No	1 (1.5%)	0 (0%)	3 (2.4%)	42 (1.8%)
**Did the household have saving? ***				
Yes	6 (9.0%)	12 (20.7%)	22 (17.3%)	434 (18.9%)
No	61 (91.0%)	45 (77.6%)	104 (81.9%)	1844 (80.4%)
Unknown	0 (0%)	1 (1.7%)	1 (0.8%)	16 (0.7%)

**Monthly saving (for who have saving) *†**	8.3 (6.1) [n=6]	9.6 (6.1) [n=12]	19.7 (22.3) [n=22]	16.8 (23.5) [n=434]
**Monthly approximate HH expenditure, Bangladeshi taka‡**	153.6 (115.2-198.7) [n=67]	146.4 (109.0-190.8) [n=58]	153.6 (115.2-197.3) [n=127]	148.8 (116.2-192.0) [n=2294]
**TCV**				
Vaccinated	22 (32.8%)	48 (84.5%)	98 (77.2%)	1838 (80.1%)
Unvaccinated	45 (67.2%)	10 (17.2%)	29 (22.8%)	456 (19.9%)
**Fever onset time at presentation, days‡**	4.0 (3.0-6.0) [n=67]	3.0 (2.0-5.0) [n=58]	3.0 (2.0-4.0) [n=127]	3.0 (2.0-4.0) [n=2294]
**Were antibiotics taken in the last 2 weeks?**				
Yes	15 (22.4%)	9 (15.5%)	23 (18.1%)	343 (15.0%)
No	52 (77.6%)	49 (84.5%)	104 (81.9%)	1944 (84.7%)
Unknown	0 (0%)	0 (0%)	0 (0%)	7 (0.3%)

[1] refers to Hindu, Christian, Buddhist, or other religions

[2] refers to toilet without flush, no toilet, or open space

[3] refers to communal tap, communal tube well, communal hand pump, water vendor, stored in reservoir, pond/canal/river, shared tap/tube well/well in household, or others

[4] refers to boiled, filtered, or chemicals treated

[#] Other pathogens include pathogens other than *S*. Typhi (including positive for *S*. Paratyphi, non-typhoidal S. and other unknow pathogens)

* Children who were from the same households were counted more than once

† mean(sd)

‡ median (IQR)

1.00 Bangladeshi Taka = 0.0096 US Dollar (the rate on 5th Jan 2023)

Supplementary Table 3. **Vaccine protection against blood-culture confirmed typhoid fever in the sensitivity analyses among all age-eligible children at any of the four vaccination campaigns during the study period**[±]

			Adjusted IRR/OR[¥]	VE (%) (95% CI)[¥]	p-value
**CRCT (150 clusters)**	**JE-recipients**	**TCV-recipients**			
	**N=33398**	**N=33822**			
Blood-culture confirmed typhoid fever (no.)/ Person-Years of follow-up	266/43480	45/43697	0.17 (0.12,0.24)	83 (76,88)	<0.0001
Incidence rate (per 100,000 PYs) (95% CI)	612 (540,690)	103 (75,138)			
**Cohort (75 TCV clusters)**	**Non-vaccinees**	**TCV-recipients**			
	**N=15770**	**N=32966**			
Blood-culture confirmed typhoid fever (no.)/ Person-Years of follow-up	53/17623	43/42978	0.33 (0.24,0.46)	67 (56,76)	<0.0001
Incidence rate (per 100,000 PYs) (95% CI)	301 (225,393)	100 (72,135)			
**Test negative design (75 TCV clusters)**	**Test negatives**[#]	**Test positives**			
Non-vaccinees	19 (23%)	90 (68%)	0.09 (0.04,0.20)	91 (80,96)	<0.0001
TCV-recipients	62 (77%)	43 (32%)			
	**Test negatives**[‡]	**Test positives**			
Non-vaccinees	65 (26%)	90 (68%)	0.05 (0.09,0.25)	85 (75,91)	<0.0001
TCV-recipients	181 (74%)	43 (32%)			
	**Test negatives**[*]	**Test positives**			
Non-vaccinees	867 (26%)	90 (68%)	0.18 (0.12,0.26)	82 (74,88)	<0.0001

TCV-recipients	2469 (74%)	43 (32%)

[±] List of all the abbreviations: CRCT cluster randomised controlled trials, CI confidence interval, IRR incidence rate ratio, OR odds ratio, PY person-year, TND test-negative case-control study design, VE vaccine efficacy;

[#] Defined as specimens that were positive for pathogens other than *S.* Typhi (excluding 165 contaminants);

[‡] Defined as specimens that were positive for pathogens other than *S.* Typhi (including 165 contaminants);

* Defined as specimens with no growth;

[¥] For CRCT and cohort analysis, IRR was adjusted for the stratifying variables for randomisation, including geographical ward, distance to study clinics (below or above median), number of eligible children at baseline, and age, sex, toilet type in the house, drinking water source, treatment of drinking water, handwashing before meals, and handwashing after defecation. For TND, OR was adjusted for age, sex, toilet type in the house, drinking water source, treatment of drinking water, handwashing before meals, handwashing after defecation, distance to study clinics (numeric), and matched calendar month of fever start date.

Supplementary Table 4. The associations between JE vaccination and blood culture-confirmed typhoid fever in the sensitivity analyses among all age-eligible children at any of the four vaccination campaigns during the study period[±]

			Adjusted IRR/OR[¥]	p-value
**Cohort (75 JE clusters)**	**Non-vaccinees**	**JE-recipients**		
	**N=15494**	**N=32616**		
Blood-culture confirmed typhoid fever (no.)/ Person-Years of follow up	49/17701	262/42824	2.20 (1.77,2.74)	<0.0001
Incidence rate (per 100,000 PYs) (95% CI)	277 (205,366)	612 (540,691)		
**Test negative design (75 JE clusters)**	**Test negatives**[#]	**Test positives**		
Non-vaccinees	19 (27%)	94 (27%)	0.85 (0.46,1.58)	0.61
JE-recipients	52 (73%)	259 (73%)		
	**Test negatives**[‡]	**Test positives**		
Non-vaccinees	82 (29%)	94 (27%)	1.14 (0.79,1.64)	0.49
JE-recipients	197 (71%)	259 (73%)		
	**Test negatives** [*]	**Test positives**		
Non-vaccinees	899 (24%)	94 (27%)	0.92 (0.72,1.19)	0.53
JE-recipients	2820 (76%)	259 (73%)		

[±] List of abbreviations: CRCT cluster randomised controlled trials, CI confidence interval, IRR incidence rate ratio, OR odds ratio, PY person-year, TND test-negative case-control study design, VE vaccine efficacy;

[#] Defined as specimens that were positive for pathogens other than *S.* Typhi (excluding 208 contaminants);

[‡] Defined as specimens that were positive for pathogens other than *S.* Typhi (including 208 contaminants);

[*] Defined as specimens with no growth;

[¥] For cohort analysis, IRR was adjusted for the stratifying variables for randomisation, including geographical ward, distance to study clinics (below or above median), number of eligible children at baseline, and age, sex, toilet type in the house, drinking water source, treatment of drinking water, handwashing before meals, and handwashing after defecation. For TND, OR was adjusted for age, sex, toilet type in the house, drinking water source, treatment of drinking water, handwashing before meals, handwashing after defecation, distance to study clinics (numeric), and matched calendar month of fever start date.

Supplementary Table 5. Associations between TCV or JE vaccination and the risk of infections by pathogens other than S.Typhi in the sensitivity analyses among all age-eligible children at any of the four vaccination campaigns during the study period±

			Adjusted IRR/OR¥	p-value
**CRCT analysis**	**JE-recipients**	**TCV-recipients**		
	**N=33398**	**N=33822**		
Blood-culture confirmed fever (no.)/ Person-Years of follow-up	56/43480	63/43697	1.14 (0.77,1.69)	0.51
Incidence rate (per 100,000 PYs) (95% CI)	129 (97,167)	144 (111,184)		
**Cohort analysis (in TCV clusters)**	**Non-vaccinees**	**TCV-recipients**		
	**N=15770**	**N=32966**		
Blood-culture confirmed fever (no.)/ Person-Years of follow-up	11/17623	62/42978	2.07(1.31,3.27)	0.0019
Incidence rate (per 100,000 PYs) (95% CI)	62 (31,112)	144 (111,185)		
**Cohort analysis (in JE clusters)**	**Non-vaccinees**	**JE-recipients**		
	**N=15494**	**N=32616**		
Blood-culture confirmed fever (no.)/ Person-Years of follow-up	11/17701	54/42824	1.86 (1.17,2.95)	0.0085
Incidence rate (per 100,000 PYs) (95% CI)	62 (31,111)	126 (95,165)		
**TND analysis (in TCV clusters)**	**Test negatives***	**Test positives#**		
Non-vaccinees	867 (26%)	19 (23%)	1.18 (0.79,2.00)	0.53
TCV-recipients	2469 (74%)	62 (77%)		
	**Test negatives***	**Test positives‡**		

Non-vaccinees	867 (26%)	65 (26%)	1.03 (0.76,1.38)	0.86
TCV-recipients	2469 (74%)	181 (74%)		
**TND analysis (in JE clusters)**	**Test negatives***	**Test positives#**		
Non-vaccinees	899 (24%)	19 (27%)	0.90 (0.52,1.54)	0.69
JE-recipients	2820 (76%)	52 (73%)		
	**Test negatives***	**Test positives‡**		
Non-vaccinees	899 (24%)	82 (29%)	0.80 (0.61,1.05)	0.11
JE-recipients	2820 (76%)	197 (71%)		

± List of abbreviations: CRCT: cluster randomised controlled trials, CI confidence interval, IRR incidence rate ratio, NCE negative control exposure, OR odds ratio, PY person-year, TND test-negative case-control study design, VE vaccine efficacy;

# Defined as specimens that were positive for pathogens other than S. Typhi (excluding 165 contaminants in TCV clusters and 208 contaminants in JE clusters);

‡ Defined as specimens that were positive for pathogens other than *S.* Typhi (including 165 contaminants in TCV clusters and 208 contaminants in JE clusters);

* Defined as specimens with no growth;

¥ For CRCT and cohort analysis, IRR was adjusted for the stratifying variables for randomisation, including geographical ward, distance to study clinics (below or above median), number of eligible children at baseline, and age, sex, toilet type in the house, drinking water source, treatment of drinking water, handwashing before meals, and handwashing after defecation. For TND, OR was adjusted for age, sex, toilet type in the house, drinking water source, treatment of drinking water, handwashing before meals, handwashing after defecation, distance to study clinics (numeric), and matched calendar month of fever start date.

Supplementary Table 6. Sensitivity TND analysis in specimens with a clinical diagnosis of typhoid among all age-eligible children at any of the four vaccination campaigns during the study period ±

			Adjusted OR¥	Vaccine protection (%) (95% CI)	p-value
**Test negative design (75 TCV clusters)**	**Test negatives#**	**Test positives**			
Non-vaccinees	13 (23%)	63 (80%)	0.04 (0.01,0.13)	96 (87,99)	<0.0001
TCV-recipients	43 (77%)	16 (20%)			
	**Test negatives‡**	**Test positives**			
Non-vaccinees	45 (28%)	63 (80%)	0.10 (0.05,0.19)	90 (81,95)	<0.0001
TCV-recipients	118 (72%)	16 (20%)			
	**Test negatives***	**Test positives**			
Non-vaccinees	464 (29%)	63 (80%)	0.10 (0.06,0.18)	90 (82,94)	<0.0001
TCV-recipients	1163 (71%)	16 (20%)			
**Test negative design (75 JE clusters)**	**Test negatives#**	**Test positives**			
Non-vaccinees	11 (28%)	63 (31%)	0.67 (0.29,1.56)		0.36
JE-recipients	29 (72%)	143 (69%)			
	**Test negatives‡**	**Test positives**			
Non-vaccinees	52 (32%)	63 (31%)	1.03 (0.65,1.65)		0.89
JE-recipients	112 (68%)	143 (69%)			
	**Test negatives***	**Test positives**			
Non-vaccinees	505 (28%)	63 (31%)	0.92 (0.67,1.27)		0.61

JE-recipients	1301 (72%)	143 (69%)

± List of abbreviations: CI confidence interval, OR odds ratio, PY person-year, TND test-negative case-control study design;

# Defined as specimens that were positive for pathogens other than *S.* Typhi (excluding 107 contaminants in TCV clusters and 124 contaminants in JE clusters);

‡ Defined as specimens that were positive for pathogens other than S. Typhi (including 107 contaminants in TCV clusters and 124 contaminants in JE clusters);

* Defined as specimens with no growth;

¥ OR was adjusted for age, sex, toilet type in the house, drinking water source, treatment of drinking water, handwashing before meals, handwashing after defecation, distance to study clinics (numeric), and matched calendar month of fever start date.

References

1. Hernán MA, Robins JM. Using Big Data to Emulate a Target Trial When a Randomized Trial Is Not Available. *Am J Epidemiol* 2016; **183**(8): 758-64.
2. Hernández-Díaz S, Huybrechts KF, Chiu YH, Yland JJ, Bateman BT, Hernán MA. Emulating a Target Trial of Interventions Initiated During Pregnancy with Healthcare Databases: The Example of COVID-19 Vaccination. *Epidemiology* 2023; **34**(2): 238-46.
3. Li G, Gerlovin H, Figueroa Muñiz MJ, et al. Comparison of the Test-negative Design and Cohort Design With Explicit Target Trial Emulation for Evaluating COVID-19 Vaccine Effectiveness. *Epidemiology* 2024; **35**(2): 137-49.
4. De Serres G, Skowronski DM, Wu XW, Ambrose CS. The test-negative design: validity, accuracy and precision of vaccine efficacy estimates compared to the gold standard of randomised placebo-controlled clinical trials. *Eurosurveillance* 2013; **18**(37): 20585.
5. Chua H, Feng S, Lewnard JA, et al. The Use of Test-negative Controls to Monitor Vaccine Effectiveness: A Systematic Review of Methodology. *Epidemiology* 2020; **31**(1): 43-64.
6. Lipsitch M, Jha A, Simonsen L. Observational studies and the difficult quest for causality: lessons from vaccine effectiveness and impact studies. *Int J Epidemiol* 2016; **45**(6): 2060-74.
7. Sanderson E, Macdonald-Wallis C, Davey Smith G. Negative control exposure studies in the presence of measurement error: implications for attempted effect estimate calibration. *Int J Epidemiol* 2018; **47**(2): 587-96.
8. Lipsitch M, Tchetgen Tchetgen E, Cohen T. Negative controls: a tool for detecting confounding and bias in observational studies. *Epidemiology* 2010; **21**(3): 383-8.
9. Lim WW, Cowling BJ, Nakafero G, Feng S, Nguyen-Van-Tam JS, Bolt H. The impact of repeated vaccination on relative influenza vaccine effectiveness among vaccinated adults in the United Kingdom. *Epidemiol Infect* 2022; **150**: e198.
10. Sullivan SG, Tchetgen Tchetgen EJ, Cowling BJ. Theoretical Basis of the Test-Negative Study Design for Assessment of Influenza Vaccine Effectiveness. *Am J Epidemiol* 2016; **184**(5): 345-53.
11. Lightowler MS, Manangazira P, Nackers F, et al. Effectiveness of typhoid conjugate vaccine in Zimbabwe used in response to an outbreak among children and young adults: A matched case control study. *Vaccine* 2022; **40**(31): 4199-210.
