# Supplementary material for: The validity of test-negative design for assessment of typhoid conjugate vaccine protection: comparison of estimates by different study designs using data from a cluster-randomised controlled trial
Source: Lancet Glob Health. 2025 Apr 16;13(6):e1122–31. doi: 10.1016/S2214-109X(25)00056-7 (PMC12095117; doi:10.1016/S2214-109X(25)00056-7)
Supplement: Equitable partnership declaration [file mmc2.pdf]

THE LANCET
Global Health

Supplementary appendix 2

This Equitable Partnership Declaration (EPD) was submitted by the authors, and we reproduce it as supplied. It has not been peer reviewed. *The Lancet*'s editorial processes have not been applied to the EPD.

Supplement to: Feng S, Zhang Y, Khanam F, et al. The validity of test-negative design for assessment of typhoid conjugate vaccine protection: comparison of estimates by different study designs using data from a cluster-randomised controlled trial. *Lancet Glob Health* 2025; published online April 16. https://doi.org/10.1016/S2214-109X(25)00056-7.

Equitable Partnership Declaration questions

Researcher considerations

1. Please detail the involvement that researchers who are based in the region(s) of study had during a) study design; b) clinical study processes, such as processing blood samples, prescribing medication, or patient recruitment; c) data interpretation; and d) manuscript preparation, commenting on all aspects. If they were not involved in any of these aspects, please explain why.

   This question is intended for international partnerships; if all your authors are based in the area of study, this question is not applicable.

   This should include a thorough description of their leadership role(s) in the study. Are local researchers named in the author list or the acknowledgements, or are they not mentioned at all (and, if not, why)? Please also describe the involvement of early career researchers based in the location of the study. Some of this information might be repeated from the Contributors section in the manuscript. Note: we adhere to ICMJE authorship criteria when deciding who should be named on a paper.

**a) Study design:**<br>This study is a re-analysis of data from the TyVAC Bangladesh cluster randomised controlled trial (CRCT) conducted in Mirpur, Dhaka, Bangladesh. Researchers from the International Centre for Diarrhoeal Disease Research, Bangladesh (icddr,b) played very important roles in all phases of the original trial and were also actively involved in this study. They contributed to discussions on the Methods for analysing vaccine effectiveness by different study designs—cohort, test-negative design, and case-control analysis, and make sure these analyses were appropriate and robust.
**b) Clinical study processes:**<br>Since this study is a re-analysis of previously collected data from the TyVAC Bangladesh CRCT, no clinical study processes were involved in this study. However, local researchers played a central role in the original TyVAC Bangladesh CRCT, leading clinical processes such as patient recruitment, vaccination campaigns, blood sample processing, laboratory analysis, and case identification. While the primary results of the CRCT have been published (DOI: 10.1016/S0140-6736(21)01124-7), this study focuses on re-analysing the data by different observational study designs to assess vaccine effectiveness.
**c) Data interpretation:**<br>Local researchers worked closely with international researchers to interpret the findings of this study. They helped in identifying potential biases in cohort study designs such as the role of healthcare-seeking behaviours.
**d) Manuscript preparation:**<br>Bangladeshi researchers were actively involved in the preparation of the manuscript, contributing to the writing and review. Their contributions were acknowledged through authorship in accordance with ICMJE criteria.

2. Were the data used in your study collected by authors named on the paper, or have they been extracted from a source such as a national survey? ie, is this a secondary analysis of data that were

not collected by the authors of this paper. If the authors of this paper were not involved in data collection, how were data interpreted with sufficient contextual knowledge?

The Lancet Global Health *believe contextual understanding is crucial for informed data analysis and interpretation.*

The data used in this study were collected from the TyVAC Bangladesh cluster randomised controlled trial (CRCT), and this study is a re-analysis of primary data collected by this CRCT using different study designs (not secondary analysis). The local and Oxford investigators, who were involved in data collection, are named on this paper and they have the contextual knowledge. Other authors contributed from an epidemiological and statistical perspective, helping to develop the study design and statistical analysis methods. This combination of local contextual knowledge and design/analytical knowledge ensured that the study was both scientifically rigorous and interpretable in the local context.

3. How was funding used to remunerate and enhance the skills of researchers and institutions based in the area(s) of study? And how was funding used to improve research infrastructure in the area of study?

   Potentially effective investments into long-term skills and opportunities within institutions could include training or mentorship in analytical techniques and manuscript writing, opportunities to lead all or specific aspects of the study, financial remuneration rather than requiring volunteers, and other professional development and educational opportunities.

   Improvements to research infrastructure could be funding of extended trial designs (such as platform trials) and use of master protocols to enable these designs, establishment of long-term contracts for research staff, building research facilities, and local control of funding allocation.

**Skills:** For this re-analysis study, researchers engaged in discussions on analytical techniques, manuscript preparation, and study design. Additionally, meetings and conferences facilitated knowledge exchange between local and international collaborators, further enhancing research skills and fostering collaboration.
**Research infrastructure:**<br>As this study was a re-analysis of available data, no additional funding was required for infrastructure development. However, the results from this study guide and facilitate the healthcare organisations to conduct future vaccine effectiveness studies.<br>Furthermore, the original study made significant contributions to research infrastructure in Bangladesh, including strengthening high-quality epidemiological surveillance and data collection,

as well as supporting laboratory facilities for blood sample collection, processing and microbiological analysis.

4. How did you safeguard the researchers who implemented the study?

Please describe how you guaranteed safe working conditions for study staff, including provision of appropriate personal protective equipment, protection from violence, and prevention of overworking.

This study was a re-analysis of available data from the TyVAC Bangladesh CRCT and did not involve any fieldwork, data collection, or in-person implementation.

Benefits to the communities and regions of study

5. How does the study address the research and policy priorities of its location?

How were the local priorities determined and then used to inform the research question? Who decided which priorities to take forward? Which elements of the study address those priorities?

This study aligns with Bangladesh's public health priorities by assessing the real-world effectiveness of the typhoid conjugate vaccine. Typhoid fever remains a major health concern, particularly in urban areas with poor sanitation and limited access to clean water. Understanding how well the vaccine works after rollout helps inform vaccine policy and public health decisions.

How were local priorities determined?
The need for this research was driven by:

- The high burden of typhoid fever in Bangladesh, making it a priority for disease prevention.
- Guidance from global health organisations like the World Health Organization (WHO) and Gavi, which emphasise evidence-based decision-making for vaccine rollouts.
- TCV will be introduced in Q2 2025 in Bangladesh.

Who decided which priorities to take forward?
The original TyVAC Bangladesh CRCT was designed in collaboration between local researchers and international experts to address key public health challenges in Bangladesh. For this re-analyses study, the research team, which included both local and international collaborators, focused on comparing different observational study designs to identify the most reliable methods for evaluating vaccine effectiveness after rollout in 2025 at Bangladesh.

Which elements of the study address those priorities?
By comparing different approaches to estimating vaccine effectiveness, this study helps inform researchers, health officials and policy makers on the reliable study design for real-world vaccine effectiveness evaluation. The findings also highlight potential biases in cohort study design, helping future studies minimise these biases when assessing vaccine effectiveness.

In summary, this study supports Bangladesh's efforts to control typhoid fever and contributes to the global discussion on vaccine effectiveness evaluation post-licensure, especially that Bangladesh is going to introduce TCV in 2025. The study provides strong mythological evidence for the future studies in monitoring VE following TCV introduction.

6. How will research products be shared in the community of study?

For instance, will you be providing written or oral layperson summaries for non-academic information sharing? Will study data be made available to institutions in the region(s) of study? The Lancet Global Health *encourages authors to translate the summary (abstract) into relevant languages after paper editing; do you intend to translate your summary?*

The findings of this study will be shared through publications and conference presentations. Results will be communicated with relevant institutions and stakeholders in Bangladesh.

We should love to have the summary translated into Bengali for local dissemination.

7. How were individuals, communities, and environments protected from harm?

a) *How did you ensure that sensitive patient data was handled safely and respectfully? Was there any potential for stigma or discrimination against participants arising from any of the procedures or outcomes of the study?*

All patient data were anonymised and handled in compliance with ethical standards to protect confidentiality. The study did not involve any procedures or outcomes that could lead to stigma or discrimination against participants.

b) *Might any of the tests be experienced as invasive or culturally insensitive?*

As this study is based on previously collected data, no additional tests were conducted.

c) *How did you determine that work was sensitive to traditions, restrictions, and considerations of all cultural and religious groups in the study population?*

The original study was designed with local researchers to ensure that all aspects respected traditions, religious practices, and cultural norms of the study population. Community engagement and ethical approvals were part of the trial process.

d) *Were biowaste and radioactive waste disposed of in accordance with local laws?*

No new biological or radioactive waste was generated in this study.

e) *Were any structures built that would have impacted members of the community or the environment (such as handwashing facilities in a public space)? If so, how did you ensure that you had appropriate community buy-in?*

No new infrastructure was built as part of this study.

f) *How might the study have impacted existing health-care resources (such as staff workloads, use of equipment that is typically employed elsewhere, or reallocation of public funds)?*

Since this study was a re-analysis of available data where data collection is not applicable, it did not affect existing health-care resources.

8. Finally, please provide the title (eg, Dr/Prof, Mr/Mrs/Ms/Mx), name, and email address of an author who can be contacted about this statement. This can be the corresponding author.

**Name:** Dr Xinxue Liu<br>**Email:** xinxue.liu@paediatrics.ox.ac.uk
